# The therapeutic effects of lacosamide on epilepsy-associated comorbidities

**DOI:** 10.3389/fneur.2023.1063703

**Published:** 2023-03-16

**Authors:** Zihua He, Jinmei Li

**Affiliations:** Department of Neurology, West China Hospital, Sichuan University, Chengdu, China

**Keywords:** epilepsy, lacosamide, anti-seizure medications, epilepsy comorbidities, psychiatric disorders

## Abstract

Epilepsy is a chronic neurological disorder associated with severe social and psychological effects, and most epilepsy patients often report at least one comorbidity. Accumulating evidence have suggested that lacosamide, a new generation of anti-seizure medications, may exhibit efficacy in the management of both epilepsy and its related comorbidities. Therefore, this narrative review aimed to elucidate the recent advancements regarding the therapeutic role of lacosamide in epilepsy-associated comorbidities. The possible pathophysiological mechanisms between epilepsy and epilepsy-associated comorbidities have been also partially described. Whether lacosamide improves cognitive and behavioral functions in patients with epilepsy has not been conclusively established. Some studies support that lacosamide may alleviate anxiety and depression in epilepsy patients. In addition, lacosamide has been found to be safe and effective in the treatment of epilepsy in people with intellectual disabilities, epilepsy of cerebrovascular etiology, and epilepsy associated with brain tumors. Moreover, lacosamide treatment has demonstrated fewer side effects on other systems. Hence, future larger and higher quality clinical studies are needed to further explore both the safety and efficacy of lacosamide in the treatment of epilepsy-associated comorbidities.

## 1. Introduction

Epilepsy is a clinically complicated syndrome and a commonly diagnosed neurological disorder. In addition to seizures, a high proportion of patients also develop comorbidities. It has been found that ~50% of adult patients with active epilepsy have comorbid conditions, including somatic as well mental comorbidities, with anxiety and depression being the most common psychiatric comorbidities. A number of previous studies have reported that about 16–23 and 18–22% of patients with epilepsy (PWE) suffered from comorbid depression and anxiety disorders, respectively, with the prevalence of the former in patients with recurrent seizures being as high as 55%. The presence of the psychiatric comorbidities can increase the recurrence, mortality, and severely affect the quality of life in PWE (1–4). Likewise, the various somatic comorbidities can complicate antiepileptic therapy and increase the medical and social burden.

Anti-seizure medications (ASMs) are currently being used as the main treatment for epilepsy. Epilepsy-associated comorbidities have a crucial impact on the treatment decisions, and some ASMs may also influence these comorbidities (5). Consequently, in clinical practice, it is necessary to screen and diagnose the different comorbidities in a timely and accurate manner. This can aid to comprehensively assess the necessity, safety, as well as feasibility of the treatment, which can facilitate the selection of appropriate treatment plan, individualize the therapy based on the etiology and significantly improve the symptoms in PWE (6, 7).

The new ASM lacosamide has been approved for the management of patients with partial-onset seizures because to its effectiveness, safety and tolerability (8–13). The seizures are primarily caused by abnormal excess or synchronization of the neuronal activity (14). The voltage-gated sodium channels (VGSCs) are activated during the membrane depolarization and play a crucial role in the generation and transmission of action potentials in the neurons by controlling the flow of sodium ions across the cell membrane (15). Abnormal activity of VGSCs has been found to closely linked with the pathophysiology of epilepsy (16). In contrast to the conventional sodium channel blocking ASMs (i.e., phenytoin and carbamazepine, etc.), it has been postulated that lacosamide can selectively increase the slow inactivation of VGSCs, thereby controlling the pathophysiological neuronal hyperexcitability by regulating the long-term availability of sodium channels (17–19). Electrophysiologic studies have shown that lacosamide can alter the slow inactivation voltage curve toward hyperpolarization and significantly accelerate the slow inactivation state of VGSCs (20–22). In addition, lacosamide can enhance the slow inactivation of VGSCs at potentials close to the resting membrane potential of the neurons, thus inhibiting action potential generation and neuronal firing (22). Moreover, another recent *in vitro* study has suggested that lacosamide can bind to fast-inactivated states of VGSCs similarly to other sodium channel blockers, but with slower binding and unbinding kinetics (23). Abnormal axon sprouting can lead to the rewiring of neuronal circuits, which can also contribute to epileptic seizures (24, 25). Collapsin response mediator protein-2 (CRMP2) is a cytoplasmic protein that is expressed mainly in neurons and oligodendrocytes, and can mediate the neuronal polarity, neurite outgrowth and axonal growth (26). Lacosamide may also regulate CRMP2 in an indirect functional interaction to prevent the formation of excitatory synaptic connections in epileptogenesis (5, 27, 28). In addition, to enhance the pharmacological effects, lacosamide can be combined with a variety of ASMs or non-ASMs due to the modest influence of drug-drug interactions during the metabolism (29). In recent years, lacosamide has also been found to have substantial positive effects on multiple epilepsy-associated comorbidities, and thus may lead to novel strategies for the clinical treatment options (3, 29).

## 2. Epilepsy-associated comorbidities

Epilepsy-associated comorbidities can increase the risk of seizures by up to tenfold, thus suggesting that epilepsy and epilepsy-associated comorbidities share diverse pathophysiological mechanisms (30). Certain comorbidities can induce epilepsy by direct or indirect mechanisms (1), and epilepsy or antiepileptic treatment might also trigger or promote some comorbidities (31). In addition, some shared risk factors or genetics can contribute to the development of epilepsy-associated comorbidities (1, 32).

### 2.1. The shared pathophysiological mechanisms between epilepsy and epilepsy-associated comorbidities

The common pathophysiological mechanisms of epilepsy and psychiatric disorders involve several complex aspects, including neurotransmitter alterations, hypothalamus-pituitary-adrenal axis (HPA) dysfunction, network/structural abnormalities, and inflammation (30). In PWE or animal models, a decrease in the monoamine neurotransmitters, such as dopamine, 5-hydroxytryptamine, and norepinephrine, can be observed (33), and deficiencies in these neurotransmitters can contribute to the development of diverse psychiatric conditions (34). Amino acid neurotransmitters, such as glutamate and gamma-aminobutyric acid (GABA), also play key roles in normal neuronal signaling, and abnormalities in glutamatergic signaling have been found to be the common pathological basis for the central nervous system related diseases (35). In preclinical experiments, dysregulation of HPA has been observed in animal models of depression as well as status epilepticus (36), and seizure-induced HPA dysfunction can increase the risk of comorbid depression (32). Comorbid psychiatric disorders have been found to be more common in frontal or temporal lobe epilepsy and are associated with abnormal network activity, including abnormalities in the hippocampus and structural changes in the amygdala (36). Disruptions of hippocampal neuron formation can directly increase the susceptibility to psychiatric disorders, while seizures can lead to altered hippocampal neuroplasticity (36, 37). Moreover, hippocampal sclerosis may affect cognition by modulating the reorganization of the cortical area connections (38). It has been reported that up to 90% of temporal lobe epilepsy patients with amygdala enlargement have comorbid depression, and amygdala volume is also closely associated with the mood disorders in PWE (39). Brain-derived neurotrophic factor (BDNF) exhibits a significant pathophysiological role in depression, and it has been suggested that BDNF might enhance seizure susceptibility by inducing the synaptic plasticity (40). Furthermore, inflammatory signaling may be involved in abnormal cerebral development, and the inflammatory factor IL-1β can contribute to epilepsy and major depression by increasing excitation (34).

### 2.2. Management of epilepsy-associated comorbidities

#### 2.2.1. Screening and evaluation for comorbid psychiatric disorders in epilepsy

Psychiatric comorbidities in PWE are often under-treated because of their late detection (7). Therefore, early identification as well as screening can facilitate prompt intervention, and provide greater medical benefit for PWE (1). The International League Against Epilepsy (ILAE) recommends using the Generalized Anxiety Disorder 7-item scale (GAD-7) as a primary screening scale. Moreover, the Hospital Anxiety and Depression Scale (HADS), the Neurologic Disorders Depression Inventory for Epilepsy (NDDI-E), and the Patient Health Questionnaire (PHQ) can also be employed to screen for anxiety or depression in PWE (7). Attention deficit hyperactivity/impulsivity disorder (ADHD) is one of the most commonly associated comorbidities in children affected with epilepsy, and the ILAE recommends the Strengths and Difficulties Questionnaire (SDQ) as a potential screening tool (level B) (41).

#### 2.2.2. Epilepsy-associated comorbidities and selection of ASMs

The objectives of treatment for epilepsy-associated comorbidities should focus on the positively controlling seizures, as well as reasonable interventions for comorbidities should be developed that can improve the patients' quality of life (4). As research progresses, it has been found that ASMs might have different psychiatric or psychological effects on patients, including the psychiatric and behavioral side effects (PBSEs) (42, 43). Some ASMs have no cognitive effects on PWE (e.g., gabapentin, and lamotrigine), whereas phenytoin sodium, topiramate, and zonisamide can adversely affect the cognition (44). In terms of the mood state, topiramate, zonisamide, and levetiracetam might have negative effects, whereas lacosamide is considered to have generally positive impact but occasionally adverse effects on the mood (44). Levetiracetam and zonisamide are associated with higher risk for PBSEs than other ASMs, although the potential mechanisms are unclear (45). In addition, there may be a cross-sensitivity between the different ASMs, i.e., one ASM causing PBSEs might simultaneously increase the risk of PBSEs associated with another ASM, which also requires attention in the clinical applications (46).

Overall, the selection of ASMs in the treatment of epilepsy-associated comorbidities need to be comprehensively considered in the benefits and risks, considering various issues such as efficacy, adverse effects, drug-drug interactions, giving priority to ASMs that can also be beneficial for the management of the comorbidities (4).

## 3. Effects of lacosamide treatment on the cognitive and behavioral functions in different populations

### 3.1. In children and adolescents

Epilepsy can increase the risk of cognitive impairment. It has been established that underlying neuropathology, seizure types, and the administration of ASMs can all have major impact on patients' cognitive abilities (47). Lacosamide is well tolerated and may have potentially positive effects on social, behavioral, and motor function while controlling seizures in children with epilepsy (48–54). Grosso et al. recruited 8 children (aged 8–16) with epileptic syndromes who had continuous spike and waves during slow sleep in a study. It was observed that after at least 12 months of follow-up, two of the five who responded to lacosamide efficacy had improved cognitive performance (55). An open label prospective study enrolled 79 children (aged 5–15) with epilepsy and evaluated the potential effect of lacosamide on behavior by Connor's Comprehensive Behavioral Rating Scales. The results indicated that adjunctive lacosamide significantly reduced the frequency of seizures and concurrently improved the patients' behavior (49). However, Farkas conducted a 16-week randomized, double-blind, placebo-controlled study to evaluate adjunctive lacosamide for the treatment of partial-onset seizures in children and adolescents. Behavioral and cognitive function were evaluated using the Achenbach Child Behavior Checklist (Achenbach CBCL) and the Behavior Rating Inventory for Executive Functioning (BRIEF). They found that behavioral and cognitive function scores were generally steady and similar in both groups (13). ADHD is a common comorbidity in children affected with epilepsy (56), and certain ASMs, such as valproic acid, possess the potential to induce or exacerbate ADHD symptoms (57, 58). Lacosamide is considered to have a possible positive or at least neutral effect on the behavioral control in ADHD, despite lack of validation of adequate evidence (59).

### 3.2. In adults

Long-term treatment with lacosamide is also well tolerated and efficacious in adult PWE, and has no significant negative effects on cognition (29, 60). However, whether it improves cognitive function remains controversial. A prospective, open-label study recruited 33 patients (aged 16–74, mean: 37 years) to evaluate the possible efficacy of lacosamide in PWE. Interestingly, lacosamide group demonstrated faster reaction times for processing the relevant information (61). Moreover, in another retrospective longitudinal study, 94 epilepsy patients were enrolled in order to compare the cognitive and behavioral effects of lacosamide and perampanel [age at first assessment: lacosamide: 40.70 (14.51), perampanel: 43.33 (11.92); mean (standard deviation)]. The Lacosamide group showed significant improvements in executive functions and memory, without a substantial increase in the self-reported aggression or irritability (60). Biton et al. pooled data from three RCTs of adjunctive lacosamide treatment for partial-onset seizures in adults. Within the permitted dose range (200 and 400 mg/day dose groups combined), the incidence of cognitive-related treatment-emergent adverse events (TEAEs) was comparable in the lacosamide and placebo groups (odds ratio: 1.3, 95% confidence interval: 0.7–2.4) and increased with lacosamide dose (62). In a prospective, open-label research with 34 adult patients with refractory epilepsy, there were no significant differences in the composite ratings of cognition or mood/quality of life before and after 6 months of treatment with lacosamide (63). In addition, several studies have demonstrated the safety and efficacy of lacosamide in elderly patients with epilepsy (64–68). However, there are currently insufficient data available regarding the cognition, mood, and quality of life of elderly patients taking lacosamide (69).

In summary, although the adverse effects of lacosamide on cognition appear to be minimal (47), it remains unclear whether it improves cognitive function in PWE. Recent experiments in animal models of epilepsy have confirmed that the strong neuroprotective effects of long-term lacosamide treatment in rats with combined neuronal damage and behavioral comorbidities (70). Future evidence from more high-quality RCTs is required to expand the in-depth understanding of this field (the details about the included clinical trials are presented in Table 1).

**Table 1:** Summaries of clinical trials on the effects of lacosamide treatment in epilepsy and psychiatric disorders.

**Authors (Ref.)**	**Participants**	***N***	**Interventions**	**Efficacy for epilepsy**	**Adverse effects of LCM**	**Effects of LCM on cognition or psychiatric disorders**
Meschede et al. (60)	Focal symptomatic epilepsy and cryptogenic epilepsy	94	Adjunctive LCM (*n* = 37) vs. Adjunctive PER (*n* = 57)	Seizure freedom: 14% (LCM group) vs.26% (PER group)	Self-perceived problems with recent memory increased (*P* = 0.024).	EpiTrack scores (*P* = 0.009) and memory performance (*P* = 0.02) improved in LCM group.
Ijff et al. (61)	Focal symptomatic epilepsy and cryptogenic epilepsy	33	Adjunctive LCM	50% of patients experienced a reduction in seizure frequency.	3 patients discontinued LCM due to side effects such as fatigue, dizziness, and ataxia.	CVST assessed information processing speed in cognition: At baseline (18.67 ± 8.9s) vs. At follow-up (15.4 ± 7.5s), *P =* 0.013.
Pasha et al. (49)	Focal refractory epilepsy	76	Adjunctive LCM	Seizure frequency was reduced by 59.9 ± 99.9%, *P* < 0.001	50.63% of patients experienced side effects such as hyperactivity, ataxia, drowsiness, and insomnia; 31.6% had hyperactivity.	Conners Comprehensive Behavior Rating Scale: At baseline (48.04 ± 10.57) vs. At follow-up (19.05 ± 05.29), *P* < 0.001.
Helmstaedter et al. (29)	epilepsy	70	Adjunctive LCM (*n* = 44) vs. Adjunctive TPM (*n* = 15) vs. Adjunctive LTG (*n* = 11)	Seizure freedom: 16% (LCM group), 13% (TPM group), 55% (LTG group)	One patient of LCM group showed significant memory decline.	23% of patients (LCM) and 27% of patients (LTG) showed a significant improvement in EpiTrack performance.
Heyman et al. (48)	Focal refractory epilepsy	17	Adjunctive LCM	A ≥50% seizure reduction was achieved in 35% patients.	Adverse effects were reported in 59% of patients, including nausea, dizziness, fatigue, etc.	Social, behavioral, and/or motor improvement was observed in 41% of patients.
Schmitz et al. (71)	Newly diagnosed focal or generalized epilepsy with psychiatric conditions	126	LCM monotherapy (*n* = 64) vs. CBZ-CR monotherapy (*n* = 62)	Seizure freedom: 6 M: 81.0 (LCM) vs. 75.6% (CBZ-CR). 12M: 62.5 (LCM) vs. 66.6% (CBZ-CR).	TEAEs (i.e., dizziness, headache, nasopharyngitis, etc.) were reported in 81.3% of LCM patients and 90.3% of CBZ-CR patients. 23.4% of LCM patients and 16.1% of CBZ-CR patients reported psychiatric TEAEs (i.e., depression, anxiety.).	NA
Rocamora et al. (3)	Focal epilepsy with symptoms of depression and anxiety	49	Adjunctive LCM	Seizure freedom: 28.3%	Adverse events: 59.2% of patients; Dizziness: 44.9% of patients; Drowsiness: 32.7% of patients.	The score of the BDI-II depression scale decreased significantly (*P* < 0.001); Pathological anxiety significantly improved both in the STAI-S/T scale (*P* = 0.011/*P* = 0.006); The HADS-A score decreased significantly in patients with “severe” anxiety levels (*P* = 0.018).
Cuomo et al. (72)	Bipolar disorder	225	LCM (*n* = 102) vs. Other ASMs (*n* = 123)	NA	Headache: 10% of patients (LCM) vs. 15% of patients (other ASMs); Dizziness: 6% of patients (LCM) vs. 8% of patients (other ASMs); Nausea: 5% of patients (LCM) vs. 18% of patients (other ASMs); Confusion and cognitive symptoms: 1% of patients (LCM) vs. 20% of patients (other ASMs).	LCM patients performed better than the control group on the YMRS, CGI-S and GAF. (*P* = 0.000147; *P* < 0.00001; *P* = 0.000521).
Nakhutina et al. (73)	Focal epilepsy	50	adjunctive LCM (*n* = 18) vs. ≥2 ASMs (*n* = 32)	Seizure freedom: 50 (LCM) vs. 34.4% (Control)	Two patients and one patient discontinued LCM due to dizziness and irritability, respectively.	POMS: Total mood distress significantly improved in the LCM group (*P* = 0.02). QOLIE-89: LCM had no significant effect on the overall quality of life (*P* = 0.078).
Giorgi et al. (74)	Focal epilepsy	10	Adjunctive LCM	Seizure frequency was reduced by 33.3%.	50% of the patients experienced mild drowsiness.	BDI and STAI-S/T scores: No significant change (*P* = 0.07; *P* = 0.08/*P* = 0.15).

LCM, Lacosamide; CBZ-CR, Carbamazepine controlled-release; TPM, Topiramate; LTG, Lamotrigine; ASMs, Anti-seizure Medications; M, Month; TEAEs, Treatment-emergent adverse events; CVST, Computerized Visual Searching Task; BDI-II, Beck's Depression Inventory-II; STAI-S/T, State–Trait Anxiety Inventory; HADS, Hospital Anxiety and Depression Scale; POMS, Profile of Mood States; QOLIE-10, Quality of Life in Epilepsy-10; QOLIE-89, Quality of Life in Epilepsy-89; YMRS, Young Mania Rating Scale; CGI-S, Clinical Global Impressions–Severity; GAF, Global Assessment of Functioning Scale; NA, Not Available.

## 4. Effects of lacosamide treatment on epilepsy-associated anxiety and depression

In recent years, researchers have explored the effects of lacosamide on anxiety and depression in PWE. A *post hoc* analysis of a large randomized controlled trial SP0993 revealed that the efficacy of lacosamide monotherapy was comparable to that of carbamazepine controlled-release (CBZ-CR) in a subgroup of epilepsy patients with comorbid psychiatric disorders, such as anxiety, depression, and insomnia (71). Moreover, in patients with partial-onset seizures, the treatment of epilepsy with lacosamide also may improv anxiety, depression as well as quality of life without worsening indicators of sleep quality and fatigue (3, 73, 74). In addition, lacosamide improved mania, anxiety, depression, and global functioning in the short term when used for the treatment of patients with bipolar disorder compared to other ASMs (72).

However, whether lacosamide can positively affect mood disorders in PWE remains controversial, with some studies suggesting that it has no significant effect on the mood or can exhibit only positively affects patients with major depressive symptoms at the baseline (75), but it is unlikely to have a negative impact and no serious safety concerns have been reported. Under standard clinical treatment conditions, the incidence of the cardiovascular and psychiatric adverse events associated with lacosamide is minimal, which was identical to the incidence of treatment discontinuation owing to adverse events (76). Therefore, it might still be a safe and effective treatment option for PWE with psychiatric comorbidities (the details of the included clinical trials are presented in Table 1).

## 5. Effects of lacosamide treatment on epilepsy patients with intellectual disability

The prevalence of the comorbid epilepsy in people with intellectual disability (PWID) ranges from 20 to 30% and increases with severity of intellectual disability (ID). It has been established that ~16% of people with epilepsy also have some degree of ID, which is significantly higher than the prevalence of <1% in the overall population (77–79). The patients with such comorbidities might exhibit more complicated neuropsychiatric characteristics that can impair the treatment outcomes, exacerbate clinical management challenges, and raise the risk of mortality (80, 81).

Brenner et al. (82) included 132 patients with refractory epilepsy in PWID in study whose primary endpoint variables were the retention rates of lacosamide which were estimated by the Kaplan-Meier method. The results showed that the retention rates were 64% at 1 year, 57% at 2 years, and 56% at 3 years, and that ID and seizure severity did not affect the continued use of the drug. However, except for a high incidence of behavior-related side effects, other adverse events were similar to those reported in the previous studies in the general population. Overall, the findings of this study concluded that lacosamide may be effective and safe for the management of epilepsy patients in PWID. In addition, another non-interventional, single-center study reported evaluating the efficacy of lacosamide in patients with ID and drug-resistant epilepsy. The results showed improved Clinical Global Impression scale in 61% of patients, with retention rates of 71 and 65% at 12 and 24 months, respectively, thus suggesting that adjunctive lacosamide may be a suitable antiepileptic treatment option for ID patients (83).

## 6. Effects of lacosamide treatment on the post-stroke epilepsy and brain tumor-related epilepsy

Stroke is a common cause of epilepsy in the elderly population, with seizure rates ranging from 3.3–3.8 to 7–14% following ischemic and hemorrhagic strokes, respectively (84–87). Seizures in the stroke patients may be caused by distinct mechanisms such as release of cytotoxic neurotransmitter leading to the neuronal hyperexcitability and deposition of gliosis and hemosiderin (88). Seizures are also prevalent in patients affected with brain tumors, with a risk of 60–100% for low-grade gliomas and 40–60% for glioblastomas (89).

According to the findings of a small observational study, 50% of elderly patients with post-stroke non-convulsive status epilepticus (NCSE) displayed controlled epileptic activity within 45–60 min of intravenous lacosamide treatment, with no recurrence and adverse effects reported over a 24-h period. This finding confirmed that lacosamide was safe and effective, thus suggesting that it has the potential to become the drug of choice for the prevention and treatment of post-stroke NCSE in the elderly (90). In a prospective study compared with the historical control group, it was concluded that the clinical efficacy of lacosamide as an add-on therapy might be superior to the historical group treated with levetiracetam in patients with brain tumor-related epilepsy, without affecting the mood and quality of life (91). Rosenow et al. (92) analyzed the efficacy of lacosamide in epilepsy patients of cerebrovascular etiology based on three large clinical studies and reported that the monotherapy efficacy of lacosamide was numerically superior to carbamazepine-CR, with a higher proportion of lacosamide patients being seizure-free after 6 and 12 months of therapy than carbamazepine-CR patients. These observations suggested that lacosamide may be useful for the treatment of epilepsy with cerebrovascular etiology.

## 7. Effects of lacosamide treatment for epilepsy on other systems

In addition to the aforementioned disorders, several common somatic comorbidities of epilepsy include the cardiovascular, musculoskeletal, respiratory, and nutritional disorders (93, 94). Traditional ASMs (e.g., carbamazepine) have a high potential for drug-drug interactions, negatively affect the lipid metabolism and can elevate the levels of various cardiac markers (95). Therefore, epilepsy-associated comorbidities can also restrict the options for epilepsy treatment (44). Among the newer ASMs, lacosamide can display relatively less interactions with other drugs, and studies have shown that it also has fewer respiratory effects, with nasopharyngitis being a frequent adverse event (7.2–17.2% occurrence) (8, 96). It has been reported that the drug does not prolong the QTc interval or adversely affect the heart rate in adult patients with partial-onset seizures and the maximum recommended dose (400 mg/day) of adjunctive lacosamide is not significantly associated with cardiac effects, except for small changes in PR interval without any major symptomatic consequences (97). It has also been found that favorable changes in the hormones and lipid levels can be observed in PWE after the lacosamide treatment (98).

## 8. Common adverse effects of lacosamide treatment

In all clinical studies, dizziness is the most common adverse effect of lacosamide (99, 100). In randomized, double-blind, placebo-controlled studies, the incidence of dizziness associated with lacosamide ranged from 23.1 to 25.9%, compared to 8–9.2% for placebo (101, 102). A 3-year follow-up of 473 PWE suggested a final dizziness rate of 26.4% (103). Another typical TEAE of lacosamide is headache. Ben-Menachem et al. (104) conducted a long-term trial of 116 patients on open-label treatment, with a median exposure duration of 854 days, and headache incidence was 9.4%. In another double-blind study with a median exposure time of 630 days, 15.1% of participants in the lacosamide group experienced headaches (96). Vision-related adverse effects such as diplopia are thought to be associated with sodium channel blockers (105). Rosenow et al. (106) recruited 376 patients and found a 13.8% incidence of diplopia over the course of 3 years. Nausea/vomiting and somnolence are also common side effect associated with lacosamide treatment (12, 107). A meta-analysis revealed that the incidence of nausea/vomiting was 9.3% (108). Long-term follow-up showed a range of 6.1% to 8.7% somnolence occurrence (96, 103). The above-mentioned frequent side events are more likely to occur during the first 3 months of treatment, and the majority are well-tolerated (100). It is noteworthy that between 0.4 and 1.7% of lacosamide-treated individuals experienced suicidal ideation or behavior (62, 101, 109). Although there is no evidence to suggest that lacosamide increases the risk of suicide-related events more than other ASMs, caution is still necessary (100).

## 9. Conclusions

The complex pathogenesis and clinical manifestations of epilepsy, as well as the propensity to acquire various comorbidities simultaneously can pose significant challenge to application of optimal therapy and increase the clinical burden. Different ASMs can also exacerbate or induce epilepsy-associated comorbidities along with their anti-epileptic effects. Consequently, numerous considerations are needed while selecting a suitable pharmacological treatment strategy. The current findings support that lacosamide may have substantial positive effects on multiple comorbidities without exhibiting serious safety concerns and may provide some reference for clinical treatment. Future studies with larger sample sizes or in specific subgroups of patients are also expected to further validate and demonstrate the influence of lacosamide on the clinical benefit in epilepsy patients with different comorbidities.

## Author contributions

ZH drafted the manuscript. JL conceptualized and designed the study and revised the manuscript. Both authors contributed to the article and approved the submitted version.
